# Genome-Wide Identification of the *MYB* and *bHLH* Families in Carnations and Expression Analysis at Different Floral Development Stages

**DOI:** 10.3390/ijms24119499

**Published:** 2023-05-30

**Authors:** Luhong Leng, Xiaoni Zhang, Weichao Liu, Zhiqiang Wu

**Affiliations:** 1 Shenzhen Branch, Guangdong Laboratory of Lingnan Modern Agriculture, Genome Analysis Laboratory of the Ministry of Agriculture and Rural Affairs, Agricultural Genomics Institute at Shenzhen, Chinese Academy of Agricultural Sciences, Shenzhen 518120, China; 82101215166@caas.cn (L.L.); lwchao2021@163.com (W.L.); 2 Kunpeng Institute of Modern Agriculture at Foshan, Shenzhen Branch, Guangdong Laboratory of Lingnan Modern Agriculture, Agricultural Genomics Institute at Shenzhen, Chinese Academy of Agricultural Sciences, Shenzhen 518124, China; zhangxiaoni@caas.cn; 3 Key Laboratory of Horticultural Plant Biology, College of Horticulture and Forestry Sciences, Huazhong Agricultural University, Wuhan 430070, China

**Keywords:** carnation, transcription factor, *MYB*, *bHLH*, anthocyanin biosynthesis, gene expression

## Abstract

Carnations are one of the most popular ornamental flowers in the world with varied flower colors that have long attracted breeders and consumers alike. The differences in carnation flower color are mainly the result of the accumulation of flavonoid compounds in the petals. Anthocyanins are a type of flavonoid compound that produce richer colors. The expression of anthocyanin biosynthetic genes is mainly regulated by *MYB* and *bHLH* transcription factors. However, these TFs have not been comprehensively reported in popular carnation cultivars. Herein, 106 *MYB* and 125 *bHLH* genes were identified in the carnation genome. Gene structure and protein motif analyses show that members of the same subgroup have similar exon/intron and motif organization. Phylogenetic analysis combining the *MYB* and *bHLH* TFs from *Arabidopsis thaliana* separates the carnation *DcaMYB*s and *DcabHLHs* into 20 subgroups each. Gene expression (RNAseq) and phylogenetic analysis shows that *DcaMYB13* in subgroup S4 and *DcabHLH125* in subgroup IIIf have similar expression patterns to those of *DFR*, *ANS,* and *GT/AT*, which regulate anthocyanin accumulation, in the coloring of carnations, and in red-flowered and white-flowered carnations, *DcaMYB13* and *DcabHLH125* are likely the key genes responsible for the formation of red petals in carnations. These results lay a foundation for the study of *MYB* and *bHLH* TFs in carnations and provide valuable information for the functional verification of these genes in studies of tissue-specific regulation of anthocyanin biosynthesis.

## 1. Introduction

Carnation (*Dianthus caryophyllus* L.), is one of the most popular cut flowers of the genus *Dianthus* in the Caryophyllaceae family. Along with roses (*Rosa* Hybrid) and chrysanthemums (*Dendranthema morifolium* (Ramat.) Tzvel.), carnations are among the most popular ornamental flowers in the world. More than 30,000 varieties of carnations have been cultivated worldwide [1], and are fancied by growers and consumers for their large flowers, numerous varieties with diverse colors, and long vase life. Flower color in carnations has long been an important target of artificial selection by plant breeders who are well aware of the economic benefits that new color varieties can yield [2]. Variations in flower color between different lineages and cultivars mainly depend upon pigment formation, with the four major pigments being chlorophyll, carotene, betaine, and flavonoids [3,4]. Differences in carnation color are mainly the result of differences in the accumulation of flavonoid compounds in petals [5].

Flavonoids are a diverse group of compounds containing pigments such as the anthocyanins, which are deposited in the vacuoles of plants, and produce colors ranging from red to purple and blue [6,7]. Anthocyanin biosynthesis is often a very complex process that involves numerous structural genes and transcription factors. These structural genes code for enzymes such as cinnamate 4-hydroxylase (*C4H*), 4-coumaroyl CoA ligase (*4CL*), phenylalanine ammonia-lyase (*PAL*), chalcone synthase (*CHS*), chalcone isomerase (*CHI*), flavanone 3-hydroxylase (*F3H*), flavonoid 3′,5′-hydroxylase (*F3′5′H*), flavonoid 3′–hydroxylase (*F3′H*), dihydroflavonol 4-reductase (*DFR*), and anthocyanidin synthase (*ANS*) [6,8]. In addition to these structural genes, transcription factors can determine flower color by regulating specific sites of anthocyanin biosynthesis. Many transcription factors are involved in anthocyanin transcription regulation, such as *R2R3-MYB*s, *bHLH*s, *WD40* (*WDR*), *WRKY*, *bZIP,* and *MADS*-box [9,10]. These transcription factors (TFs) regulate anthocyanin structural gene expression mainly by binding cis-acting elements in downstream structural gene promoters. Among them, the MYB–bHLH–WD40 (MBW) complex is the most widely studied TF regulating the anthocyanin synthesis pathway [11,12,13].

The MBW complex includes three types of regulatory proteins, including the R2R3-MYBs, bHLHs, and WD40 proteins. The MYB family is one of the largest families of transcription factors in plants, and is important in the regulation of anthocyanin biosynthesis [14,15]. According to the number of R (arginine) motifs in the *MYB* gene, they can be divided into four subfamilies, from *1R-MYB* with one R domain, to *4R-MYB* with four R domains. Of the four subfamilies, *R2R3-MYB* is the most abundant and widely involved in regulating plant growth and development [16,17,18]. The bHLH family is also a large class of transcription factors in plants. The *bHLH* transcription factors regulate many cellular processes, such as the fate of epidermal cells, photomorphogenesis, and flower organ development [19]. The *bHLH* transcription factor has been shown to be involved in anthocyanin biosynthesis in many plants [20,21,22,23]. The first 200 amino acids (MIR) of the N-terminal of the protein sequence of *bHLH* are regions that interact with *MYB*, and the next 200 amino acids usually contain negatively charged regions necessary for interaction with *WD40* or the RNA poly Ⅱ complex. *WD40* is not usually catalytic but serves as a docking platform for many interacting proteins [7,24]. In *Arabidopsis thaliana*, three regulatory proteins, namely, AtTT2/AtMYB123, AtTT8/AtbHLH04, and AtTTG1 (WD40-protein), have been reported to act together as MBW complexes to promote proanthocyanidin production in the seed coat by activating target gene *BAN/ANR* expression [25,26]. Two kinds of MYB (DcMYB6 and DcMYB7) proteins have been shown to interact with members of the MBW complex to control anthocyanin biosynthesis in purple carrots (*Daucus carota var. sativa* Hoffm.) [12]. Study in lychee (*Litchi chinensis* Sonn.) has found that *LcbHLH1* and *LcbHLH3* play an important role in *LcMYB1*’s regulation of anthocyanin production, suggesting that the *LcMYB1–LcbHLH* complex enhances anthocyanin accumulation and may be related to the activation of *DFR* and *ANS* transcription [27].

In this study, we performed genome-wide identification of *DcaMYB* and *DcabHLH* genes, and a total of 106 *MYB* genes and 125 *bHLH* genes were identified from the carnation genome. The gene structure, motif distribution, chromosomal location, phylogeny, and the expression of genes at each flowering stage were comprehensively analyzed. From these analyses, this study aims to comprehensively understand *MYB* and *bHLH* genes in carnations and reveal their roles in regulating anthocyanin synthesis as it relates to flower color. These results provide information for further functional analysis of *DcaMYBs* and *DcbHLHs,* ultimately improving the breeding and development of new color varieties in carnations.

## 2. Results

### 2.1. Identification of DcaMYBs and DcabHLHs

We identified 106 *MYB* genes and 125 *bHLH* genes in the carnation genome, combining the results of homologous, conserved motif, and HMM identification. The *MYB* and *bHLH* genes from carnation were named *DcaMYB1-DcaMYB106* and *DcabHLH1-DcabHLH125*, separately. Further sequence analysis shows that *DcaMYBs* and *DcabHLHs* products differ in both amino acid sequence and molecular weight. The longest *MYB* gene is *DcaMYB51,* with 1027 amino acids, and the shortest *DcaMYB73* gene only has 136 amino acids; the longest *bHLH* gene is *DcabHLH21,* with 969 amino acids and *DcabHLH55* is the shortest at only 90 amino acids. The molecular weights of the *DcaMYBs* range from 34,425.05 to 251,683.56, with isoelectric points ranging between 4.85 (*DcaMYB93*) and 5.22 (*DcaMYB92*). Similarly, the molecular weights of the *DcabHLHs* range from 21,838.14 to 237,109.03, with isoelectric points between 4.87 (*DcabHLH21*, *DcabHLH112*) and 5.35 (*DcabHLH55*). The main sequence information and molecular weight and isoelectric point of the predicted protein are given in Supplementary Materials Tables S1 and S2.

### 2.2. Phylogenetic Analyses of Carnation MYBs and bHLHs with A. thaliana

**Figure 1:**
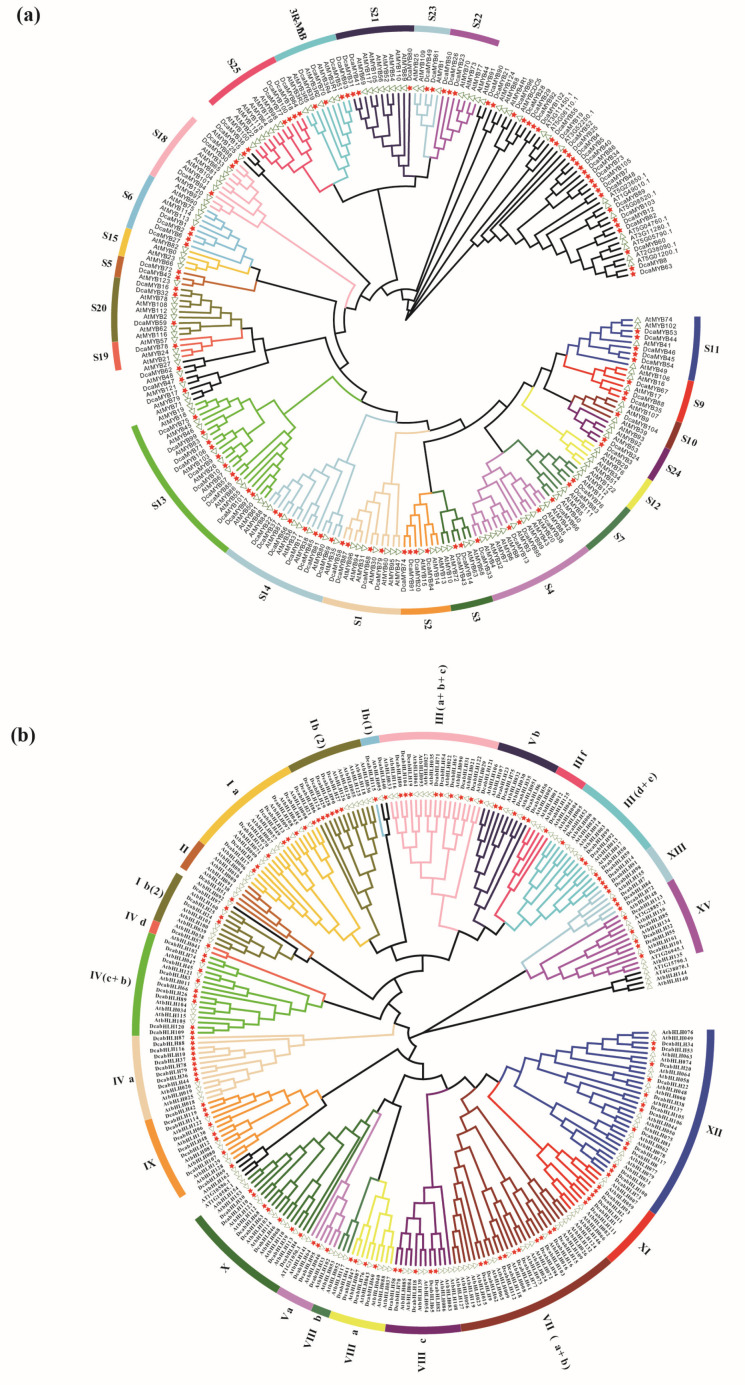
Phylogenetic analysis of *MYB* and *bHLH* proteins from carnation and *A. thaliana*. (**a**) Phylogenetic analysis of *MYB* proteins from carnation and *A. thaliana*. (**b**) Phylogenetic analysis of *bHLH* proteins from carnation and *A. thaliana*. Different colored arcs represent different subgroups of the domain as denoted from preestablished names in *A. thaliana*. The red five-pointed star and the green triangle represent carnation and *A. thaliana* terminals, respectively.

The phylogenetic trees of *MYB*s and *bHLH*s were constructed separately using full-length amino acids sequences from carnation and *A. thaliana*. After removing alternative splicing in the *A. thaliana* genes, 142 *MYB*s and 153 *bHLH*s sequences from *A. thaliana* (Tables S3 and S4), and 106 *MYB*s and 125 *bHLH*s sequences from carnation were assessed using neighbor-joining (NJ) trees (Figure 1). *DcaMYB*s are divided into 21 gene subgroups (S1, S2 S3, S4, S5, S6, S7, S9, S10, S11, S13, S14, S18, S19, S20, S21, S22, S23, S25, and 3R-MYB) according to the classification of *AtMYBs* (Figure 1a). Unlike the *A. thaliana* MYB family [28,29], which is divided into 23 subfamilies, the subfamilies S12 and S15 are not found in carnation. Among the subfamilies, the S13 subfamily has the most *DcaMYBs* genes (including *DcaMYB9, 10*, *57*, *71*, *75*, *85*, *97*, *99*, *101*, and *106*), while only a single *DcaMYB* gene is found in the S10 (*DcaMYB104*) and S19 subfamilies (*DcaMYB78*). Similarly, *DcabHLHs* are divided into 21 subgroups according to the grouping of the NJ tree with *A. thaliana* (Figure 1b), while in *A. thaliana*, the bHLH family is divided into 22 subgroups [19,30,31], with no carnation genes found in subgroup VIIIa. The Xll subgroups contain 13 *DcabHLH* genes (including *DcabHLH5*, *8*, *20*, *22*, *34*, *38*, *53*, *73*, *81*, *100*, *105*, *106*, and *117*), which makes them the subgroups with the largest number of genes. Only a single *DcaMYB* gene is found in the Ⅳd, Ⅱ, and Ⅲf subgroups (*DcabHLH57*, *DcabHLH51*, and *DcabHLH125*) of carnation.

### 2.3. Gene Structure and Motif Analysis of DcaMYBs and DcabHLHs

**Figure 2:**
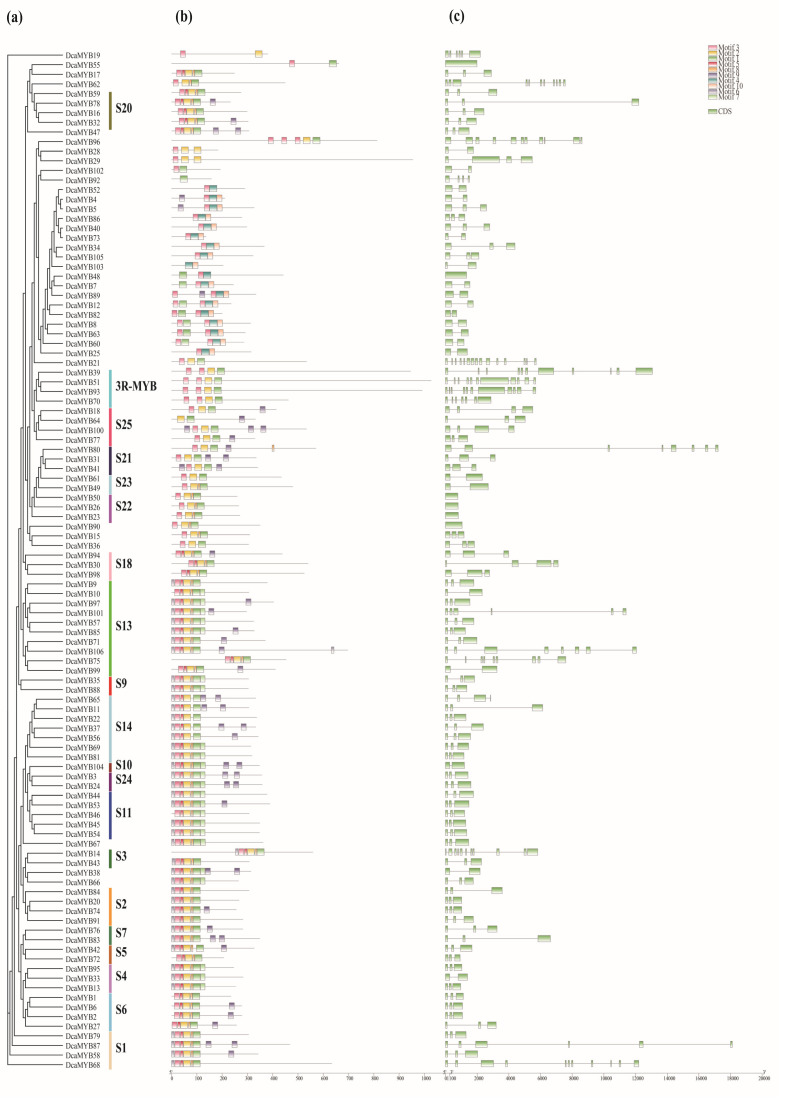
Phylogenetic relationships, motif distribution, and gene structure of *MYBs* in carnation. (**a**) Phylogenetic analysis of 106 *DcaMYB*s of carnation. Different subgroups are marked with different colored vertical lines. (**b**) Schematic diagram of *MYB* conserved protein motifs. Different colored boxes represent different protein motifs, and black lines represent non-conserved sequences. (**c**) The exon/intron structure of different *MYB* genes. Green boxes represent exons, the lines between the boxes are introns.

**Figure 3:**
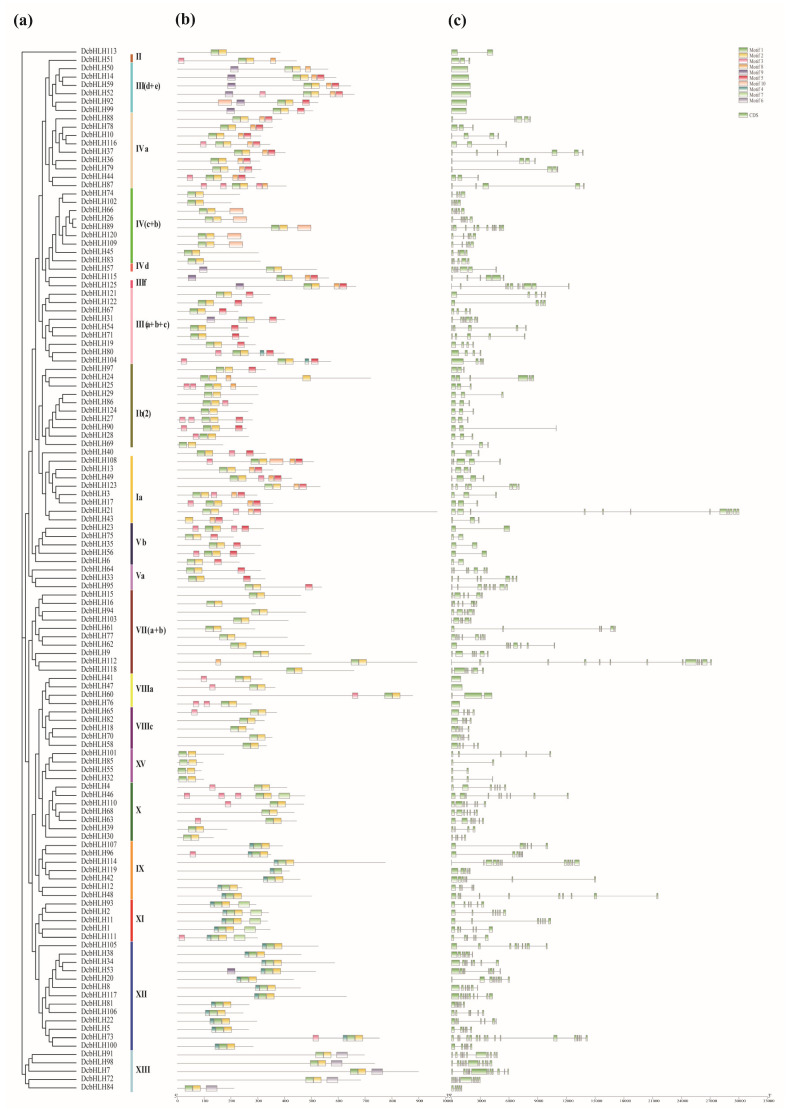
Phylogenetic relationship, motif distribution, and gene structure of *bHLHs* in the carnation genome. (**a**) Phylogenetic analysis of 125 *bHLH*s. Different subgroups are marked with different colored vertical lines. (**b**) Schematic diagram of bHLH conserved protein motifs. Different colored boxes represent different motifs, and black lines represent non-conserved sequences. (**c**) The exon/intron structure of different *bHLH* genes. Green boxes represent exons, the lines between the boxes are introns.

In this study, 10 conserved motifs of *DcaMYB* and *DcabHLH* transcription factors were separately identified using MEME. Most motifs are found located in an ordered fashion at the N terminal of *DcaMYB*s, while in only a few cases, the motifs are distributed irregularly at the C terminal. Motif 1, 2, 3, 5, 6, and 9 are widely distributed in the domain of *DcaMYB*s. A few motifs, such as motif 4 and motif 10, cluster together on a distinct branch of the phylogenetic tree (Figure 2b and Figure S1). We identify 10 conserved motifs in *DcabHLH*s and find that all protein sequences contain conserved motifs 1 and 2 (Figure 3b and Figure S2). The same subgroups usually contain similar motifs, for example, motif 6 only exists in the ⅩⅢ subgroup. Motif 10 is only found in subgroup Ⅳ (c + b), and, similarly, motif 9 is mainly concentrated in subgroup Ⅲ (d + e).

All introns and exons are identified in *MYB* and *bHLH* genes in carnation. Most genes in the same subgroup have exons and introns of similar length and number (Figure 2c and Figure 3c). For example, all the genes in the S22 subfamily of *MYB* contain no introns and are roughly the same length. Similarly, all the genes in the Ⅲ (d + e) subgroup of *bHLH* contain no introns. In general, the motif composition and gene structure of *MYB* and *bHLH* genes of the same group are similar. The phylogenetic analysis results strongly support the reliability of the group classifications derived from similarity to known *A. thaliana* subgroups.

### 2.4. Chromosomal Locations of DcaMYBs and DcabHLHs

**Figure 4:**
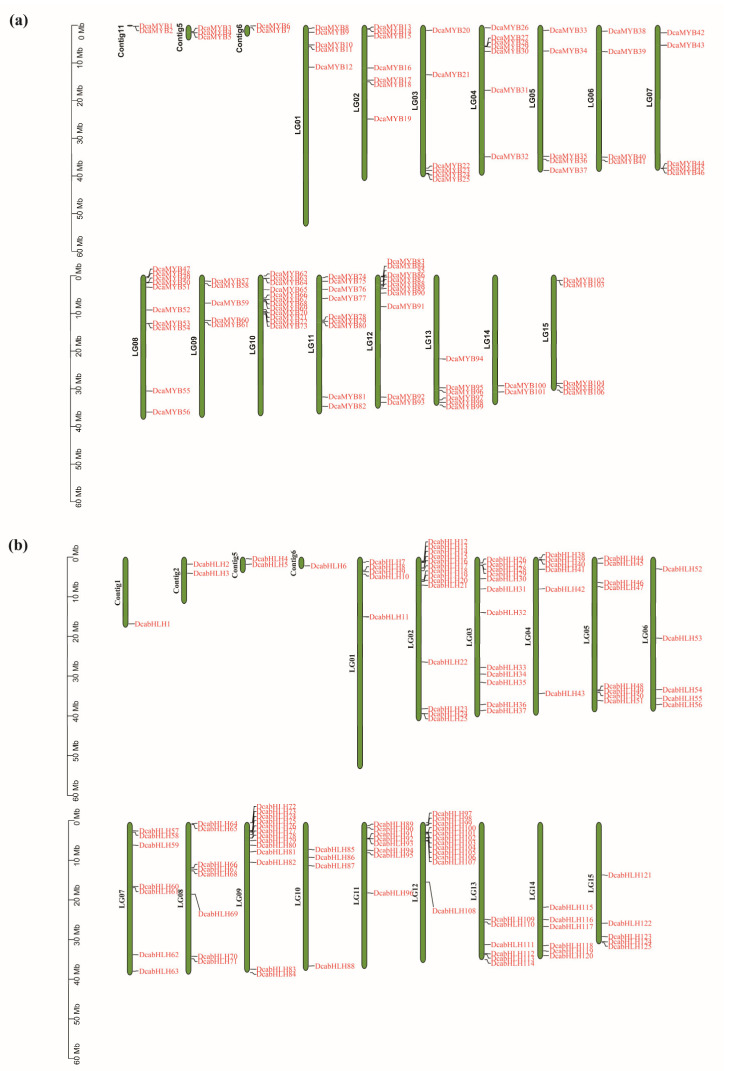
Chromosome location mapping. (**a**) Chromosome locations of *DcaMYBs*. (**b**) Chromosome locations of *DcabHLHs*. Chromosome numbers are shown on the left side of each chromosome. The scale is in megabases (Mb). Each gene is named according to the order of its corresponding position on a chromosome.

Most *DcaMYBs* are unevenly distributed on 15 chromosomes, and seven are mapped to three unanchored contigs. Of the 125 *DcabHLHs,* 119 are unevenly distributed on 15 chromosomes, and 6 are mapped to four unanchored contigs (Figure 4). In the *MYB* gene family of carnations, chromosome 10 has the most *DcaMYBs* at 12, chromosome 12 has 11, chromosome 8 has 10, and in all other chromosomes with *MYB* genes, they each have between one and nine genes. The collinearity analysis reveals 21 pairs of segmental duplicated genes in the MYB family of carnations, with the largest number of segmental duplicated genes found on chromosome 13 (Figure S3). In the *bHLH* gene family of carnations, chromosome 2 has the most *DcabHLHs* at 14, chromosome 9 has 13, chromosome 3 and 12 have 12, and in all other chromosomes with *bHLH* genes, they each have between one and nine genes. The collinearity analysis reveals 31 pairs of segmental repeat genes in the bHLH family of carnations, with the largest number of segmental repeat genes found on chromosome 3 (Figure S3).

### 2.5. Expression Profiles of DcaMYBs and DcabHLHs

**Figure 5:**
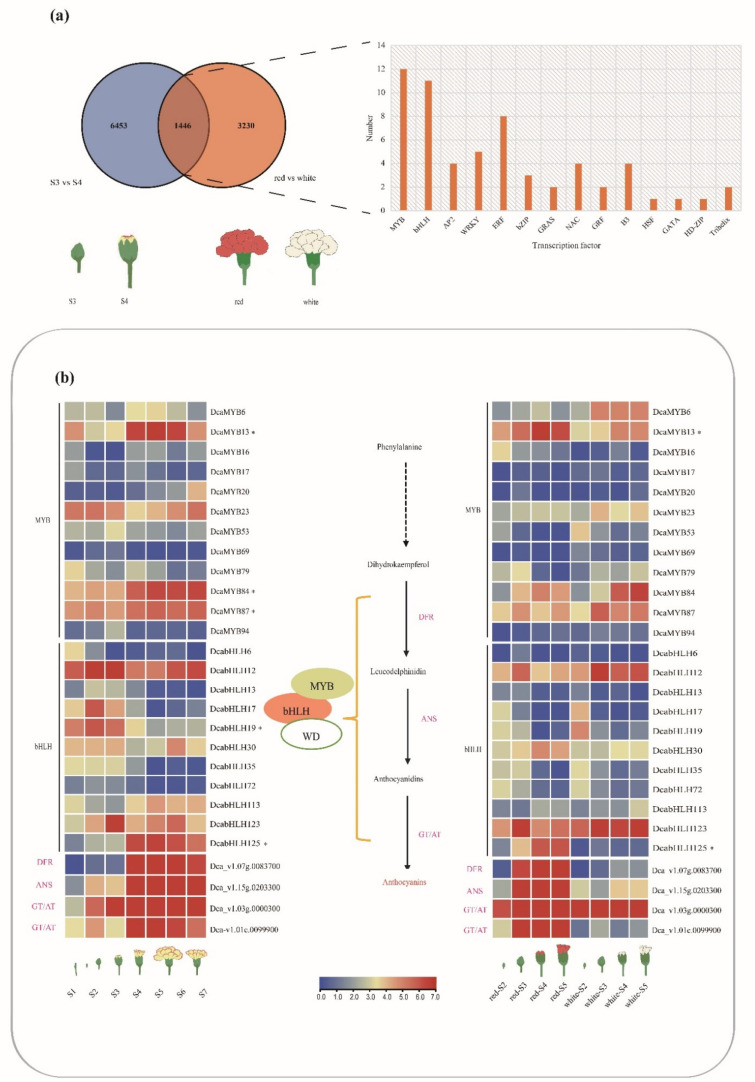
Correlation analysis of *DcaMYBs* and *DcabHLHs* with the formation of red petals in carnations. (**a**) Venn diagram shows the number of differentially expressed genes between the two comparisons (S3 vs. S4 and red vs. white). (**b**) Expression of *DcaMYB* and *DcabHLH* in carnations. Dotted arrows indicate multi-step process, asterisk indicates a gene putatively involved with differences in color. S1: the axillary bud stage, the stem tip meristem begins to transform into floral primordium; S2: floral organogenesis; S3: completely closed flower bud; S4: flowers beginning to color; S5: half-blooming flowers; S6: fully open flowers; and S7: senescing flowers.

In the process of evolution, different genes in a family often diverge functionally over time, sometimes resulting in new functions among genes. Gene expression studies are an important means by which such divergence in gene function can be detected. In order to explore the synthesis of anthocyanins regulated by *MYB* and *bHLH* transcription factors in carnations, we analyzed the two groups of differentially expressed genes representing floral color variation (S3 vs. S4 and red vs. white), S3 and S4 referring to the third and fourth stages of flower development, where S3 indicates uncolored flowers and S4 indicates flowers starting to color, as well as red-flowered carnations and white-flowered carnations at full bloom (Figure 5a). We identified a total of 1446 genes that overlapped between the two groups. These include various transcription factors such as *MYB*, *bHLH*, *AP2*, *WRKY,* and *bZIP*, of which there are 12 *MYB* transcription factors and 11 *bHLH* transcription factors (Figure 5a).

To further understand the role of these *MYB* and *bHLH* genes during the color formation of carnation petals, we analyzed their expression in seven floral development stages (S1–S3 petals are colorless and S4–S7 petals have red margins), and in four floral stages of red flower and white flower of carnations. Previous studies show that genes such as *DFR*, *ANS*, and *GT/AT* also affect anthocyanin biosynthesis [32,33]. In our study, the *DFR*, *ANS*, and *GT/AT* genes in carnations are identified and their expressions increase with petal coloring (Figure 5). We find that *DcaMYB13* and *DcabHLH125* not only have similar expression patterns, but they also share similar expression patterns with *DFR*, *ANS*, and *GT/AT* in the process of petal coloring of seven floral development stages. In addition, they show the same trend in four stages of red flower development, but not in the white flower. We also find that the expression pattern of *DcaMYB84* and *DcaMYB87* are similar to that of *DFR*, *ANS,* and *GT/AT* in the seven floral development stages with petal coloring. *DcabHLH19* is highly expressed during the S1–S3 non-colored stage, but decreases during the S4–S7 stage when red floral margin coloration occurs, presumably as a negative regulator (Figure 5b).

## 3. Discussion

Carnations are one of the most popular ornamental flowers in the world, with diverse colors and beautiful patterns as one of the key characteristics attracting growers and consumers [34,35,36]. Differences in carnation flower color is the result of many factors, with anthocyanin pigment deposition in petal cells being one of the main factors [2,37]. In ornamental plants studied thus far, *MYB* transcription factors have been found to be important in the biosynthesis of anthocyanin, as well as *bHLH* transcription factors, which can interact with *MYB*-producing proteins to regulate anthocyanin biosynthesis [13,38,39].

In this study, genome-wide analysis was used to explore the regulation of carnation flower color by the MYB and bHLH families. Based on conserved features, genes are grouped into different subgroups. A large number of studies show that the genes related to regulating anthocyanin biosynthesis in *MYB* are mainly concentrated in the S4, S5, S6, and S7 subgroups [18,29]. Among them, the *MYB* genes of the S5, S6, and S7 subgroups mainly function as activators to promote anthocyanin biosynthesis [28,40]. In the S6 subgroup, genes such as *AtMYB75*, *AtMYB113*, *AtMYB114* in *A. thaliana* [41,42], *MdMYB10* [43], *MdMYB110a* [44] in apple (*Malus pumila* Mill.), *VvMYBA1* [45], and *VvMYBA2* [46] in grape (*Vitis vinifera* L.) have been shown to positively promote anthocyanin synthesis. However, the *MYB* genes related to anthocyanin synthesis in the S4 subgroup function as repressors to inhibit anthocyanin biosynthesis [47,48,49]. In carnations, four *DcaMYB* genes (*DcaMYB13*, *33*, *38*, and *95*) are apparent in the S4 subgroup, whereas in A. thaliana, eleven *AtMYB* genes (*AtMYB3*, *4*, *6*, *7*, *8*, *20*, *32*, *42*, *43*, *85*, and *99*) are known. Similarly, the S7 subgroup in carnations has fewer genes than in A. thaliana, which may be the result of gene loss in carnation evolution. This suggests that the carnation MYB family may have undergone neofunctionalization and sub-functionalization. Most of the *bHLH* genes known from the Ⅲf subgroup, such as *AtbHLH1*, *AtbHLH2*, and *AtbHLH42*, are reported to be involved in anthocyanin biosynthesis [19,30,31]. *DcabHLH125* is clustered in subgroup Ⅲf (Figure 1b) and its expression increases with petal coloring, suggesting that this gene is involved in regulating anthocyanin biosynthesis in carnations.

In addition, we analyzed the protein motif content and gene structure of *MYB* and *bHLH* gene families in carnations. Most motifs of *R2R3-MYB* in *DcaMYB*s are found to be distributed at the N-terminal, while the conserved DNA-binding domain at the N terminus determines the binding of MYB to different acting elements, with a few of these motifs irregularly distributed at the C terminal. Motifs 1, 2, and 3 are present in almost all *DcaMYB*s. However, some motifs only appear in specific groups, such as motif 4 and motif 10, which only occur in *MYB*-related gene families, suggesting a specific function for these motifs (Figure 2b). Similar findings are found in *DcabHLH*s proteins where motifs 1 and 2 are found in all proteins, while motif 6 is only present in subgroup ⅩⅢ and motif 9 is concentrated in subgroup Ⅲ (d + e) (Figure 3b). Most *DcaMYB*s and *DcabHLH*s contain two exons and one intron, but some genes are found to be made up of only one exon. In general, protein motif and gene structure are similar within the same subgroup.

In the MYB transcription factors, it has been reported that there are multiple genes involved in regulating anthocyanin biosynthesis from the S4, S5, S6, and S7 subgroups [39]. In our study, expression analysis shows that *DcaMYB13* belongs to the S4 subgroup, and its expression pattern is similar to that of *DFR*, *ANS*, and *GT/AT*, which regulate anthocyanin accumulation, suggesting that *DcaMYB13* is likely to be involved in anthocyanin biosynthesis. The expression of *DcaMYB84* and *DcaMYB87* during the seven flower development stages in carnation is also similar to that of genes such as *DFR*, suggesting that they may be candidates for the regulation of anthocyanin synthesis. We also reviewed the literature related to the bHLH family and found that genes in subgroup Ⅲf are often involved in anthocyanin biosynthesis [30,31]. We find that *DcabHLH125* belongs to subgroup IIIf and its expression pattern is similar to that of *DFR*, *ANS*, and *GT/AT,* which indicates that it may play an important role in anthocyanin synthesis in carnation. *DcabHLH19* is highly expressed during the S1–S3 non-colored stage but decreases during the S4–S7 stage when red coloration occurs, suggesting that it may act as a negative regulator, inhibiting anthocyanin biosynthesis.

The biosynthesis of anthocyanins is regulated by the transcription factors in the WBD complex [50]. In the model species *A. thaliana*, *AtTT8* (*bHLH*) regulates anthocyanin synthesis by forming MBW transcription complexes with *AtTT2* (*MYB*) and *AtTTG1* (*WDR*). The interaction of *AN2* (*MYB*) and *JAF13* (*bHLH*) is found to promote pigment accumulation in petunias (*Petunia spp.*) [38]. The co-expression of *MdMYB10* with *MdbHLH3* and *MdbHLH33* in apples can effectively induce anthocyanin biosynthesis [43]. In our study, *DcaMYB13* and *DcabHLH125* have similar expression patterns in transcriptomes, and they are similar to those of *DFR*, *ANS,* and *GT/AT,* suggesting that *DcaMYB13* and *DcabHLH125* may interact and jointly regulate the expression of the downstream genes *DFR*, *ANS,* or *GT/AT* to promote anthocyanin biosynthesis. Further elucidating the interaction between *MYB* and *bHLH* gene families in the biosynthesis of anthocyanin in carnation flowers will be directly applicable to the development of novel cultivars. To these ends, we have begun a series of gene-editing experiments to better understand protein interactions between these gene families.

## 4. Materials and Methods

### 4.1. Identification of MYB and bHLH Family Members in the Carnation Genome

Based on the carnation genome [51] assembled by our team at the Shenzhen Genome Institute of the Chinese Academy of Agricultural Sciences in 2022, the potential *MYB* and *bHLH* genes were identified. The 168 *MYB* protein sequences and 225 *bHLH* protein sequences of *A. thaliana*, as well as 138 *MYB* protein sequences and 211 *bHLH* protein sequences of rice (*Oryza sativa* L.), were downloaded from the Plant Transcription Factor Database [52,53] as target sequences. BLASTP (BLAST 2.12.0+) were performed using these amino acid sequences above as queries against the carnation genome database. All candidate MYB and bHLH family sequences were confirmed with the NCBI conserved domain database (https://www.ncbi.nlm.nih.gov/Structure/cdd/wrpsb.cgi, accessed on 1 July 2022). The hidden Markov model (HMM) file of the *MYB* DNA-binding domain (PF00249) and the *bHLH* DNA-binding domain (PF00010) from the Pfam database (http://pfam.xfam.org/search, accessed on 3 July 2022) were used to further identify *MYB* and *bHLH* transcription factors by HMM search in TBtools [54].

### 4.2. Phylogenetic Analysis and Classification of Carnation DcaMYBs and DcabHLHs

The full-length amino acid sequences of *MYB*s and *bHLH*s derived from *A*. *thaliana* and carnation were used for phylogenetic analysis. An unrooted neighbor-joining (NJ) tree was constructed using MEGA11 with bootstrap test of 1000 replicates to assess clade support [55]. The carnation *DcaMYBs* and *DcabHLHs* were classified into different groups according to how they clustered with known *MYBs* and *bHLHs* from *A. thaliana* [19,29,30]. Finally, the phylogenetic tree was rendered in Evolview [56].

### 4.3. Gene Structure and Conserved Motif Analysis of DcaMYBs and DcabHLHs

Motif Elicitation (MEME) (http://memesuite.org/tools/meme, accessed on 13 July 2022) was used to confirm conserved motifs in *DcaMYB*s and *DcabHLH*s protein sequences. The parameters settings used in MEME were as follows: the maximum number of motifs was set to 10, while the other parameters were kept at default. TBtools was used to visualize the results.

### 4.4. Chromosomal Locations of DcaMYBs and DcabHLHs

The chromosomal locations of *DcaMYBs* and *DcabHLHs* in the carnation genome was obtained by TBtools according to the annotation data of the carnation genome. From this, the *MYB* and *bHLH* gene families were named according to their positions on chromosomes.

### 4.5. RNA-Sequencing (RNA-seq) Data Analysis of DcaMYBs and DcabHLHs

In order to better characterize the changing flower color of carnation, the transcriptome data of samples at seven flower development stages of carnation were used to find differences in expression (PRJNA796118). In addition, transcriptome data of red and white carnation petals separately at four stages of petal development were downloaded [57]. Clean reads were obtained by removing adapter-containing reads, poly-N-containing reads, and low-quality reads from the raw data. Using Hisat2 2.1.0 software, we mapped the clean reads to the reference genome publish in 2022 [51] using default parameters. The output of the mapping was processed with String Tie to obtain FPKM (Fragments Per Kilobase of exon model per Million mapped fragments) for all genes in each sample. TBtools software was used to generate the heatmap of expression (average FPKM values) at different stages.

## Data Availability

Genome assembly and annotations and RNA-seq were deposited in the NCBI BioProject under accessions PRJNA796118.

## Supplementary Materials

The following supporting information can be downloaded at: https://www.mdpi.com/article/10.3390/ijms24119499/s1.

## References

[1] İNce A.G., Karaca M. (2015). Td-DAMD-PCR assays for fingerprinting of commercial carnations. Turk. J. Biol..

[2] Morimoto H., Ando Y., Sugihara H., Narumi-Kawasaki T., Takamura T., Fukai S. (2021). Information on Flower Coloration and Pigmentation in Current Carnation Cultivars for Use in Future Flower-color Breeding. Hortic. J..

[3] Iijima L., Kishimoto S., Ohmiya A., Yagi M., Okamoto E., Miyahara T., Tsujimoto T., Ozeki Y., Uchiyama N., Hakamatsuka T. (2020). Esterified carotenoids are synthesized in petals of carnation (*Dianthus caryophyllus*) and accumulate in differentiated chromoplasts. Sci. Rep..

[4] Tanaka Y., Sasaki N., Ohmiya A. (2008). Biosynthesis of plant pigments: Anthocyanins, betalains and carotenoids. Plant J..

[5] Nakayama M. (2020). Flower Pigments Responsible for Cyanic, Yellow, and Cream-White Coloration in Carnation. The Carnation Genome.

[6] Belwal T., Singh G., Jeandet P., Pandey A., Giri L., Ramola S., Bhatt I.D., Venskutonis P.R., Georgiev M.I., Clement C. (2020). Anthocyanins, multi-functional natural products of industrial relevance: Recent biotechnological advances. Biotechnol. Adv..

[7] Pourcel L., Irani N.G., Lu Y., Riedl K., Schwartz S., Grotewold E. (2010). The formation of Anthocyanic Vacuolar Inclusions in *Arabidopsis thaliana* and implications for the sequestration of anthocyanin pigments. Mol. Plant.

[8] Liao X., Ye Y., Zhang X., Peng D., Hou M., Fu G., Tan J., Zhao J.-L., Jiang R., Xu Y. (2022). The genomic and bulked segregant analysis of Curcuma alismatifolia revealed its diverse bract pigmentation. aBIOTECH.

[9] Albert N.W., Davies K.M., Lewis D.H., Zhang H., Montefiori M., Brendolise C., Boase M.R., Ngo H., Jameson P.E., Schwinn K.E. (2014). A conserved network of transcriptional activators and repressors regulates anthocyanin pigmentation in eudicots. Plant Cell.

[10] Moglia A., Florio F.E., Iacopino S., Guerrieri A., Milani A.M., Comino C., Barchi L., Marengo A., Cagliero C., Rubiolo P. (2020). Identification of a new R3 MYB type repressor and functional characterization of the members of the MBW transcriptional complex involved in anthocyanin biosynthesis in eggplant (*S. melongena* L.). PLoS ONE.

[11] Lloyd A., Brockman A., Aguirre L., Campbell A., Bean A., Cantero A., Gonzalez A. (2017). Advances in the MYB-bHLH-WD Repeat (MBW) Pigment Regulatory Model: Addition of a WRKY Factor and Co-option of an Anthocyanin MYB for Betalain Regulation. Plant Cell Physiol..

[12] Xu Z.S., Yang Q.Q., Feng K., Xiong A.S. (2019). Changing Carrot Color: Insertions in DcMYB7 Alter the Regulation of Anthocyanin Biosynthesis and Modification. Plant Physiol..

[13] Xu W., Dubos C., Lepiniec L. (2015). Transcriptional control of flavonoid biosynthesis by MYB-bHLH-WDR complexes. Trends Plant Sci..

[14] Pattanaik S., Xie C.H., Yuan L. (2008). The interaction domains of the plant Myc-like bHLH transcription factors can regulate the transactivation strength. Planta.

[15] Yan H., Pei X., Zhang H., Li X., Zhang X., Zhao M., Chiang V.L., Sederoff R.R., Zhao X. (2021). MYB-Mediated Regulation of Anthocyanin Biosynthesis. Int. J. Mol. Sci..

[16] Chen Z., Wu Z., Dong W., Liu S., Tian L., Li J., Du H. (2022). MYB Transcription Factors Becoming Mainstream in Plant Roots. Int. J. Mol. Sci..

[17] Thakur S., Vasudev P.G. (2022). MYB transcription factors and their role in Medicinal plants. Mol. Biol. Rep..

[18] Cao Y., Li K., Li Y., Zhao X., Wang L. (2020). MYB Transcription Factors as Regulators of Secondary Metabolism in Plants. Biology.

[19] Hao Y., Zong X., Ren P., Qian Y., Fu A. (2021). Basic Helix-Loop-Helix (bHLH) Transcription Factors Regulate a Wide Range of Functions in Arabidopsis. Int. J. Mol. Sci..

[20] Verma D., Jalmi S.K., Bhagat P.K., Verma N., Sinha A.K. (2020). A bHLH transcription factor, MYC2, imparts salt intolerance by regulating proline biosynthesis in Arabidopsis. FEBS J..

[21] Jia N., Wang J.J., Liu J., Jiang J., Sun J., Yan P., Sun Y., Wan P., Ye W., Fan B. (2021). DcTT8, a bHLH transcription factor, regulates anthocyanin biosynthesis in Dendrobium candidum. Plant Physiol. Biochem..

[22] Tao R., Yu W., Gao Y., Ni J., Yin L., Zhang X., Li H., Wang D., Bai S., Teng Y. (2020). Light-Induced Basic/Helix-Loop-Helix64 Enhances Anthocyanin Biosynthesis and Undergoes CONSTITUTIVELY PHOTOMORPHOGENIC1-Mediated Degradation in Pear. Plant Physiol..

[23] Li M., Sun L., Gu H., Cheng D., Guo X., Chen R., Wu Z., Jiang J., Fan X., Chen J. (2021). Genome-wide characterization and analysis of bHLH transcription factors related to anthocyanin biosynthesis in spine grapes (*Vitis davidii*). Sci. Rep..

[24] Kelemen Z., Sebastian A., Xu W., Grain D., Salsac F., Avon A., Berger N., Tran J., Dubreucq B., Lurin C. (2015). Analysis of the DNA-Binding Activities of the Arabidopsis R2R3-MYB Transcription Factor Family by One-Hybrid Experiments in Yeast. PLoS ONE.

[25] Kleindt C.K., Stracke R., Mehrtens F., Weisshaar B. (2010). Expression analysis of flavonoid biosynthesis genes during *Arabidopsis thaliana* silique and seed development with a primary focus on the proanthocyanidin biosynthetic pathway. BMC Res. Notes.

[26] Lepiniec L., Debeaujon I., Routaboul J.M., Baudry A., Pourcel L., Nesi N., Caboche M. (2006). Genetics and biochemistry of seed flavonoids. Annu. Rev. Plant Biol..

[27] Lai B., Du L.N., Rui L., Bing H., Su W.B., Qin Y.H., Zhao J.T., Wang H.C., Hu G.B. (2016). Two LcbHLH Transcription Factors Interacting with LcMYB1 in Regulating Late Structural Genes of Anthocyanin Biosynthesis in Nicotiana and Litchi chinensis During Anthocyanin Accumulation. Front. Plant Sci..

[28] Stracke R., Werber M., Weisshaar B. (2001). The R2R3-MYB gene family in *Arabidopsis thaliana*. Curr. Opin. Plant Biol..

[29] Dubos C., Stracke R., Grotewold E., Weisshaar B., Martin C., Lepiniec L. (2010). MYB transcription factors in Arabidopsis. Trends Plant Sci..

[30] Toledo-Ortiz G., Huq E., Quail P.H. (2003). The Arabidopsis basic/helix-loop-helix transcription factor family. Plant Cell.

[31] Pires N., Dolan L. (2010). Origin and diversification of basic-helix-loop-helix proteins in plants. Mol. Biol. Evol..

[32] Morimoto H., Narumi-Kawasaki T., Takamura T., Fukai S. (2019). Analysis of Flower Color Variation in Carnation (*Dianthus caryophyllus* L.) Cultivars Derived from Continuous Bud Mutations. Hortic. J..

[33] Ozeki Y., Iijima L., Higuchi K., Miyahara T., Sasaki N., Tsujimoto T., Abe Y., Matsuba Y., Nishizaki Y., Suzuki-Wagner A. (2020). Molecular Mechanisms of Carnation Flower Colors via Anthocyanin and Flavonoid Biosynthetic Pathways. The Carnation Genome.

[34] Yagi M., Kosugi S., Hirakawa H., Ohmiya A., Tanase K., Harada T., Kishimoto K., Nakayama M., Ichimura K., Onozaki T. (2014). Sequence analysis of the genome of carnation (*Dianthus caryophyllus* L.). DNA Res..

[35] Lou X., Anwar M., Wang Y., Zhang H., Ding J. (2020). Impact of inorganic salts on vase life and postharvest qualities of the cut flower of Perpetual Carnation. Braz. J. Biol..

[36] Onozaki T. (2018). Breeding of carnations (*Dianthus caryophyllus* L.) for long vase life. Breed. Sci..

[37] Liang Q., Jin Y., Zhu Q.-H., Shao D., Wang X., Ma X., Liu F., Zhang X., Li Y., Sun J. (2023). A MYB transcription factor containing fragment introgressed from *Gossypium bickii* confers pink flower on *Gossypium hirsutum* L. Ind. Crops Prod..

[38] Quattrocchio F., Wing J.F., van der Woude K., Mol J.N., Koes R. (1998). Analysis of bHLH and MYB domain proteins: Species-specific regulatory differences are caused by divergent evolution of target anthocyanin genes. Plant J..

[39] Feller A., Machemer K., Braun E.L., Grotewold E. (2011). Evolutionary and comparative analysis of MYB and bHLH plant transcription factors. Plant J..

[40] Liu J., Osbourn A., Ma P. (2015). MYB Transcription Factors as Regulators of Phenylpropanoid Metabolism in Plants. Mol. Plant.

[41] Zhang Y., Yan Y.P., Wang Z.Z. (2010). The Arabidopsis PAP1 Transcription Factor Plays an Important Role in the Enrichment of Phenolic Acids in Salvia miltiorrhiza. J. Agric. Food Chem..

[42] Gonzalez A., Zhao M., Leavitt J.M., Lloyd A.M. (2008). Regulation of the anthocyanin biosynthetic pathway by the TTG1/bHLH/Myb transcriptional complex in Arabidopsis seedlings. Plant J..

[43] Espley R.V., Hellens R.P., Putterill J., Stevenson D.E., Kutty-Amma S., Allan A.C. (2007). Red colouration in apple fruit is due to the activity of the MYB transcription factor, MdMYB10. Plant J..

[44] Chagné D., Lin-Wang K., Espley R.V., Volz R.K., How N.M., Rouse S., Brendolise C., Carlisle C.M., Kumar S., Silva N.D. (2012). An Ancient Duplication of Apple MYB Transcription Factors Is Responsible for Novel Red Fruit-Flesh Phenotypes. Am. Soc. Plant Biol..

[45] Kobayashi S., Goto-Yamamoto N., Hirochika H. (2005). Association of VvmybA1 Gene Expression with Anthocyanin Production in Grape (*Vitis vinifera*) Skin-color Mutants. J. Jpn. Soc. Hortic. Sci..

[46] Kobayashi S., Goto-Yamamoto N., Hirochika H. (2004). Retrotransposon-Induced Mutations in Grape Skin Color. Science.

[47] LaFountain A.M., Yuan Y.W. (2021). Repressors of anthocyanin biosynthesis. New Phytol..

[48] Chen L., Hu B., Qin Y., Hu G., Zhao J. (2019). Advance of the negative regulation of anthocyanin biosynthesis by MYB transcription factors. Plant Physiol. Biochem..

[49] Aharoni A., De Vos C.H., Wein M., Sun Z., Greco R., Kroon A., Mol J.N., O’Connell A.P. (2001). The strawberry FaMYB1 transcription factor suppresses anthocyanin and flavonol accumulation in transgenic tobacco. Plant J..

[50] Liu Y., Ma K., Qi Y., Lv G., Ren X., Liu Z., Ma F. (2021). Transcriptional Regulation of Anthocyanin Synthesis by MYB-bHLH-WDR Complexes in Kiwifruit (*Actinidia chinensis*). J. Agric. Food Chem..

[51] Zhang X., Lin S., Peng D., Wu Q., Liao X., Xiang K., Wang Z., Tembrock L.R., Bendahmane M., Bao M. (2022). Integrated multi-omic data and analyses reveal the pathways underlying key ornamental traits in carnation flowers. Plant Biotechnol. J..

[52] Tian F., Yang D.-C., Meng Y.-Q., Jin J., Gao G. (2019). PlantRegMap: Charting functional regulatory maps in plants. Nucleic Acids Res..

[53] Katiyar A., Smita S., Lenka S.K., Rajwanshi R., Chinnusamy V., Bansal K.C. (2012). Genome-wide classification and expression analysis of MYB transcription factor families in rice and Arabidopsis. BMC Genom..

[54] Chen C., Chen H., Zhang Y., Thomas H.R., Frank M.H., He Y., Xia R. (2020). TBtools: An Integrative Toolkit Developed for Interactive Analyses of Big Biological Data. Mol. Plant.

[55] Tamura K., Stecher G., Kumar S., Battistuzzi F.U. (2021). MEGA11: Molecular Evolutionary Genetics Analysis Version 11. Mol. Biol. Evol..

[56] Subramanian B., Gao S., Lercher M.J., Hu S., Chen W.-H. (2019). Evolview v3: A webserver for visualization, annotation, and management of phylogenetic trees. Nucleic Acids Res..

[57] Totsuka A., Okamoto E., Miyahara T., Kouno T., Cano E.A., Sasaki N., Watanabe A., Tasaki K., Nishihara M., Ozeki Y. (2018). Repressed expression of a gene for a basic helix-loop-helix protein causes a white flower phenotype in carnation. Breed Sci..

