# Supplementary material for: Genome-Wide Identification of the MYB and bHLH Families in Carnations and Expression Analysis at Different Floral Development Stages
Source: Int J Mol Sci. 2023 May 30;24(11):9499. doi: 10.3390/ijms24119499 (PMC10254004; doi:10.3390/ijms24119499)
Supplement: Supplementary file 1 [file ijms-24-09499-s001.zip › Figure S3. Schematic representation of the chromosome distribution and interchromosomal relationships of the MYB and bHLH genes in carnation.pdf]

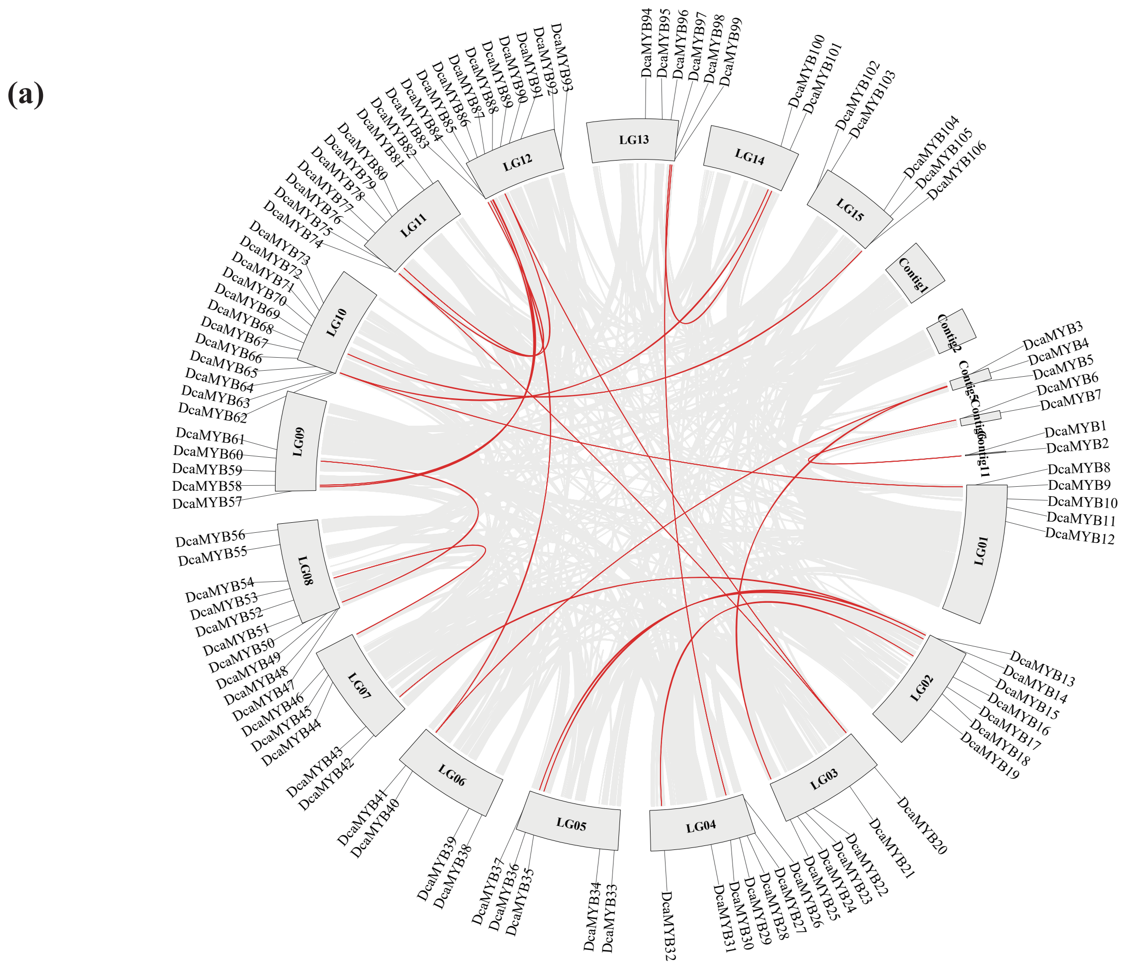

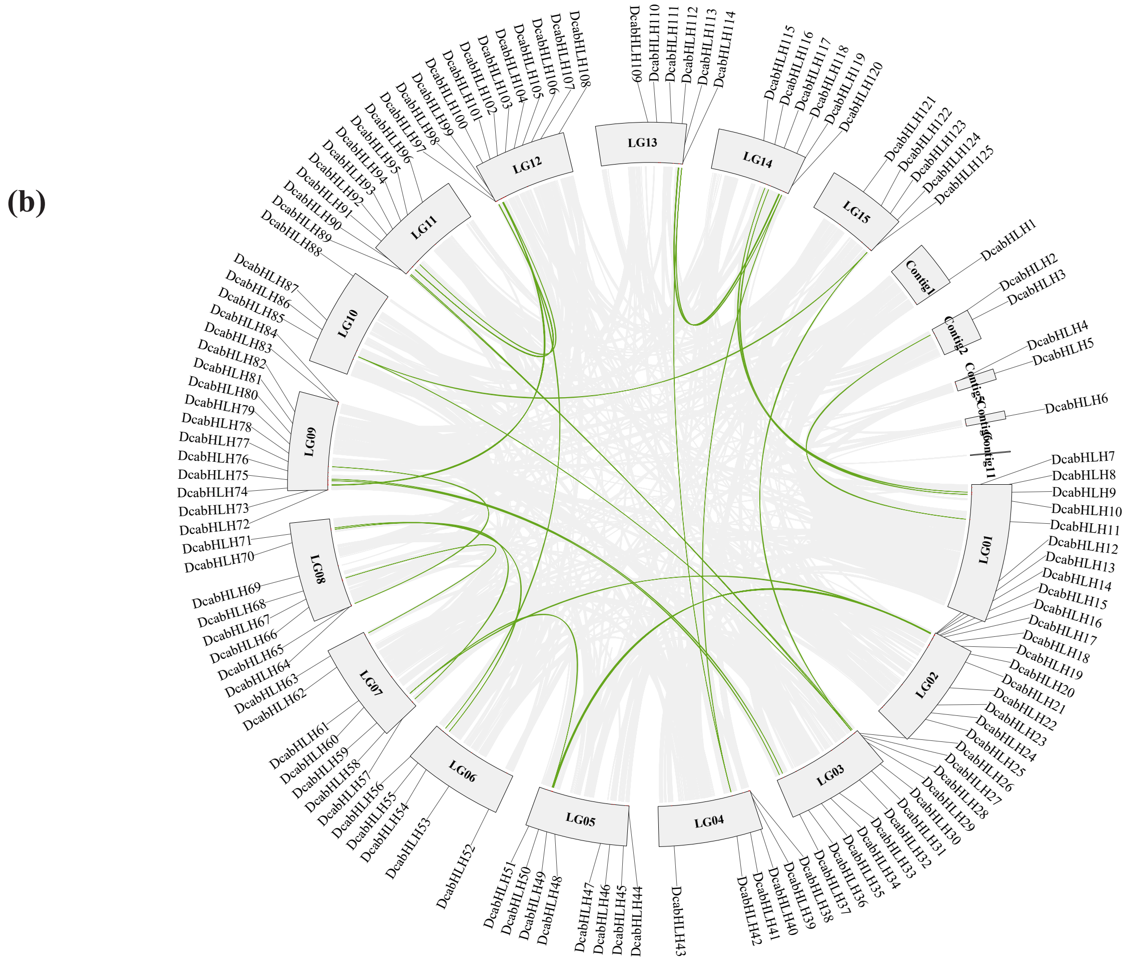

Schematic representation of the chromosome distribution and interchromosomal relationships of the MYB and bHLH genes in carnation. The gray line indicates all homozygous blocks in the Carnation genome, the red line indicates duplicated MYB gene pairs, and the green indicates duplicated bHLH gene pairs. Chromosome numbers are shown in the middle of each chromosome.
