# Supplementary figures and images for: Genome-Wide Identification of the MYB and bHLH Families in Carnations and Expression Analysis at Different Floral Development Stages
Source: Int J Mol Sci. 2023 May 30;24(11):9499. doi: 10.3390/ijms24119499 (PMC10254004; doi:10.3390/ijms24119499)

motif 1

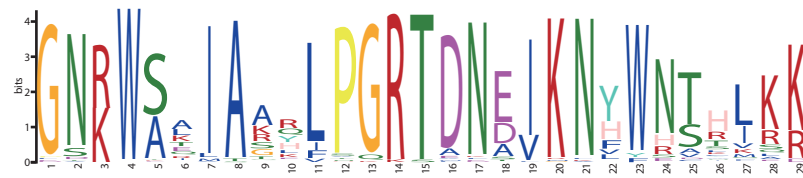

motif 2

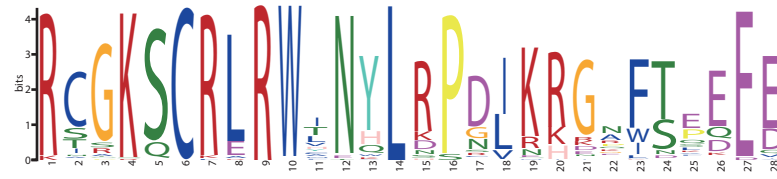

motif 3

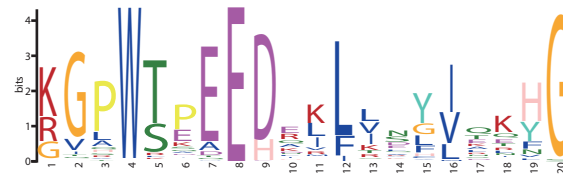

motif 4

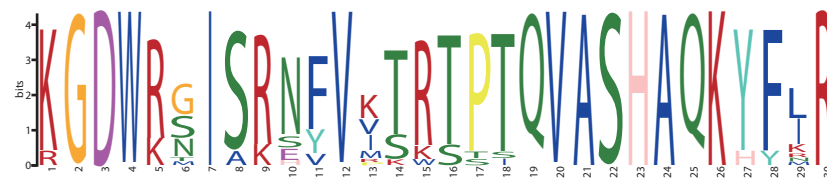

motif 5

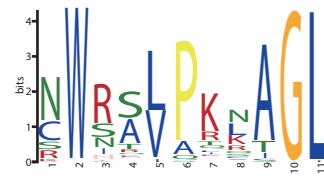

motif 6

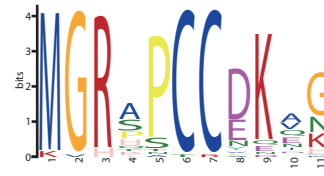

motif 7

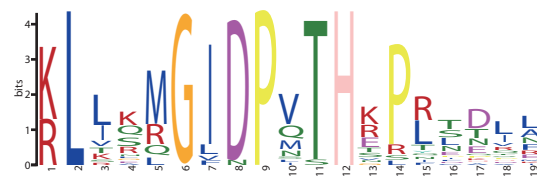

motif 8

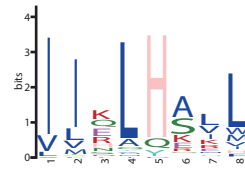

motif 9

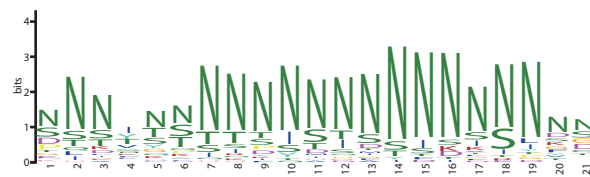

motif 10

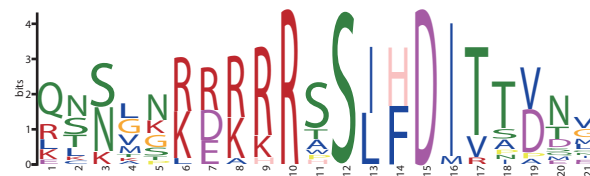

Supplement: Supplementary file 1 [file ijms-24-09499-s001.zip › Figure S1. The sequence logos of 10 conserved motifs in DcaMYB genes were analyzed with the MEME program..pdf]

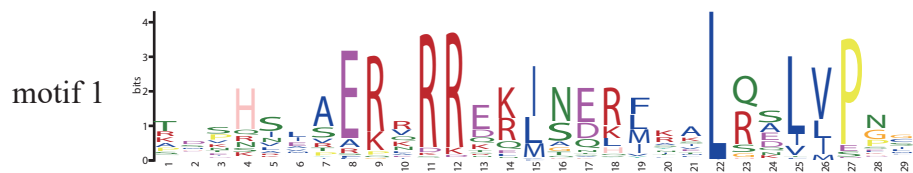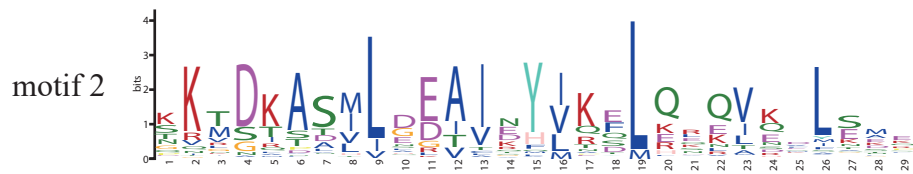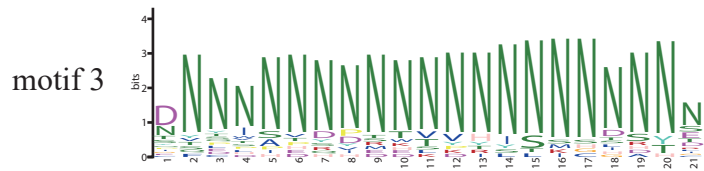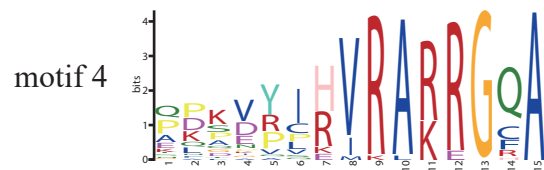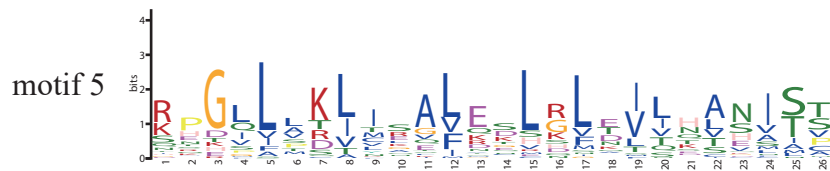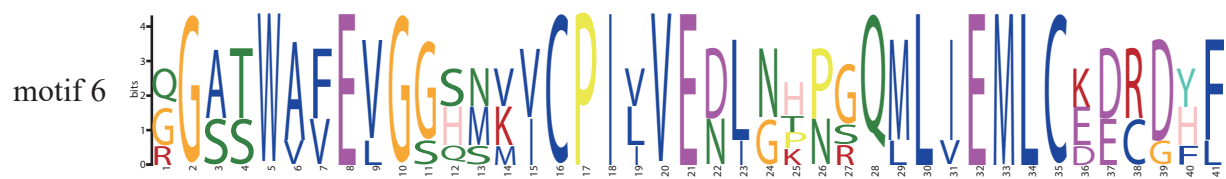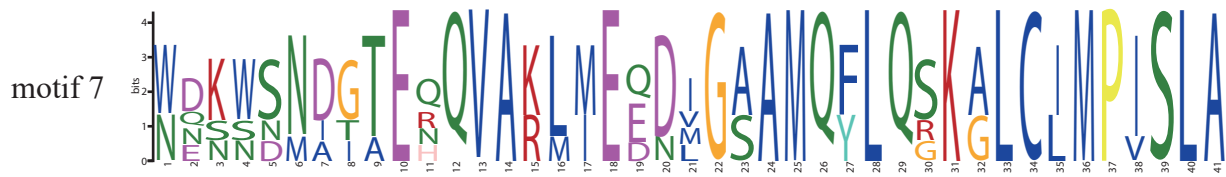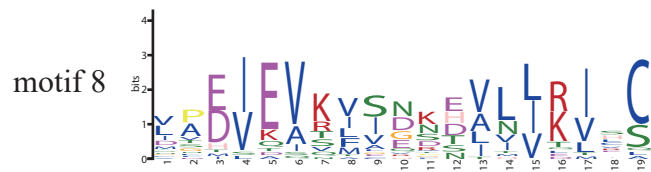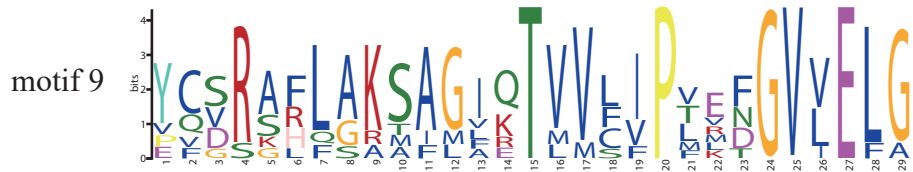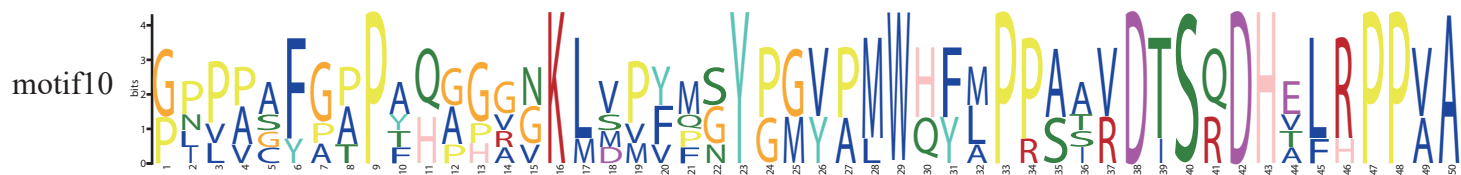

Supplement: Supplementary file 1 [file ijms-24-09499-s001.zip › Figure S2. The sequence logos of 10 conserved motifs in DcabHLH genes were analyzed with the MEME program.pdf]
